# The Cost of Provisions

**Published:** 1894-09-29

**Authors:** 


### The Institutional Workshop.

### HOSPITAL FINANCE.

### X.—THE COST OF PROVISIONS.

The variation shown in the various hospitals with regard to the expenditure on provisions must awake a feeling of astonishment in the most experienced observer of such phenomena. A good housekeeper of the old-fashioned sort who, like Mrs. Carlyle, understood the far-reaching consequences of an extra farthing on each loaf, could always furnish an estimate within a very close margin of the cost of feeding a given number of persons in a year. And it seems reasonable to expect a certain general accord, at least among hospitals of the same size, the same description, in the same town, with regard to their butchers' and bakers' bills, and no less reasonable to count upon the average cost of provisions per bed remaining about the same from year to year. A very slight inspection of the published reports suffices to show that no uniform rate of expenditure in either respect exists.

For example: the average number of beds occupied at King's, University and Westminster in 1892 was practically the same, from 183 to 186; the average cost of provisions, however, on these occupied beds was £18 at University, £23 at Westminster, and £26 at King's.

Again; comparing one year with another we find the Charing Cross Hospital reduced the expenditure on provisions from £29 in 1891 to £25 in 1892, the number of occupied beds remaining about the same. The London, on the other hand, with an increase of 18 occupied beds in 1892, increased the expenditure on each bed for provisions by £2 10s.

It would seem indeed that some spirit of malignity in hospital statistics impels them to work out with a view to defeat sound calculations and confound the most cherished maxims of common sense.

That the cost per head of provisioning a household diminishes in proportion as the numbers increase, is a familiar experience to all housekeepers; but in hospital finance we find two of the largest general hospitals, the London with 10,070 in-patients, and St. Bartholomew's with 5,528, spending respectively £28 10s. and £29 on each occupied bed for provisions, while hospitals treating about a quarter of the number of patients, such as University and the Royal Free, average from £6 to £11 less. In special hospitals the discrepancy is equally marked. The Evelina Hospital for Children, with an average of 51 occupied beds, spends £30 a year on each for provisions, while the North-Eastern Hospital for Children, with an average of but 41 occupied beds, spends no less than £10 less on each. The Samaritan Free Hospital for Women and Children, with an average of 50 occupied beds, spends £4 a year more on each for provisions than the Grosvenor, with an average of only 13.

Again, where tea, sugar, and butter are provided by the patient himself, some reduction of cost might confidently be looked for, but the cost of provisions at St. Thomas's and Westminster, where the patients provide nothing, is from £3 to £5 lower than at the London, Middlesex, and King's, where this regulation is in force. Let anyone take the trouble to reckon the cost of providing two or three hundred people daily with these articles of food, and he will see that a very material reduction should appear in hospitals where this cost is not incurred. And if such a regulation, objectionable in theory and troublesome in practice, has not economy to recommend it, on what grounds is it still retained?

Turning from London to the provinces the same amazing difference in the cost of food is apparent, ranging from the Chesterfield and North Derbyshire Infirmary, where the cost of provisions on each occupied bed is £14, or 6·92d. per day, to the £30 attained by the Durham County and the Hartlepool Hospital on a similar number of patients. The following table shows the average cost of provisions on each occupied bed for the various classes of general hospitals, and the extreme between which the average falls. Special hospitals present so wide a variation in size and treatment that it is impossible to compare them *en gros*, and they are therefore omitted.

Cost of Provisions per Occupied Bed.

| | No. of Hospitals. | Average. | Highest. | Lowest. |
|---|---|---|---|---|
| London Clinical ... ... ... | 13 | £26 | St. Mary's, £32 ... ... | University £18. |
| London Non-Clinical... ... ... | 11 | £27 | Metropolitan, £37 ... ... | West London, £18. |
| Provincial Clinical ... ... ... | 11 | £$20\frac{3}{4}$ | Radcliffe Infirmary, £26 ... | Birmingham General, £18. Birmingham Queen's, £18. Liverpool Royal Southern, £18. |
| Provincial Non-Clinical ... ... | 57 | £$21\frac{1}{3}$ | Saffron Walden, £31 ... | Chesterfield, £14. |
| Scotch and Irish Clinical ... ... | 7 | £19 | Adelaide, Dublin, £23 ... | Aberdeen Royal, £16. |
| Scotch and Irish Non-Clinical ... | 13 | £18 | Perth County, £24 ... ... | Arbroath Infirmary, £14. |

It would be no easy task to account for the remarkable difference in the cost of provisions apparent in the above table. There are, indeed, not sufficient data on which to ground a probable opinion, for no statistics relating to the feeding of an institution can be of real value unless the number of persons to be catered for is clearly defined, and a mere average of occupied beds affords but the roughest estimate of the actual household. The medical staff, the nursing staff and home, the ward servants, the kitchen, the laundry, the out-door servants—the number and requirements in all these departments differ in almost every hospital, and the numerous variations which they present from the victualling point of view are sufficient in themselves to account for a wide difference in cost between one hospital and another. A brief statement in the annual report of the numbers included in the household under these various headings would no doubt remove much misconception on this point and justify in many instances variations of expenditure hitherto inexplicable. For example, an abnormally low average for provisions might be shown to proceed from no niggardliness towards the patients, but from the payment of board wages in lieu of board to certain of the employés, while an increase in the average might well arise from the necessity of keeping an extra staff of nurses in readiness for some emergency. Until more precise information is forthcoming on the domestic management of the hospitals, any attempt to fix a working average of reasonable expenditure on food must be hopeless. As the hospitals fall more and more under popular support, the expediency of affording data for appreciating their domestic economy will become gradually apparent, and the day may not be far distant when any hospital whose published accounts show a marked variation from the average arrived at by common experience, shall feel bound to render in the ordinary course of business routine a full explanation of the cause.

